# Selenium and Taurine Combination Is Better Than Alone in Protecting Lipopolysaccharide-Induced Mammary Inflammatory Lesions via Activating PI3K/Akt/mTOR Signaling Pathway by Scavenging Intracellular ROS

**DOI:** 10.1155/2021/5048375

**Published:** 2021-12-13

**Authors:** Dandan Liu, Jiashan Lin, Wenmiao He, Kehe Huang

**Affiliations:** ^1^College of Veterinary Medicine, Nanjing Agricultural University, Nanjing, 210095 Jiangsu Province, China; ^2^Institute of Animal Nutritional Health, Nanjing Agricultural University, Nanjing, 210095 Jiangsu Province, China

## Abstract

Mastitis is mainly induced by gram-negative bacterial infections, causing devastating economic losses to the global cattle industry. Both selenium (Se) and taurine (Tau) exhibit multiple biological effects, including reducing inflammation. However, no studies have reported the protective effect of the combined use of Se and Tau against mastitis, and the underlying mechanisms remain unclear. In this study, lipopolysaccharide (LPS), the vital virulence factor of gram-negative bacteria, was used to construct the *in vivo* and *vitro* mastitis models. The results of *in vivo* model showed that Se and Tau combination was more effective than either substance alone in reducing tissue hyperemia, edema, and neutrophil infiltration in the mammary acinar cavity, improving the blood-milk barrier in LPS-induced mice mastitis, and decreasing the expression of proinflammatory factors and the activity of MPO. Moreover, Se and Tau combination significantly increased the levels of LPS-induced reduction in PI3K/Akt/mTOR, but the expressions of TLRs and NLRP3 were not significantly changed in the mammary tissue. In the *in vitro* experiments, the effects of Se and Tau combination or alone on inflammatory factors, inflammatory mediators, MPO activity, and blood-milk barrier were consistent with those *in vivo*. The Se and Tau combination has also been found to increase the survival rate of BMECs compared with each substance alone via promoting cellular proliferation and inhibiting apoptosis. Also, it has been confirmed that this combination could restore the LPS-induced inhibition in the PI3K/Akt/mTOR signaling pathway. Inhibition of mTOR by Rapamycin counteracted the combined protection of SeMet and Tau against LPS-induced inflammatory damage, the inhibition of PI3K by LY294002 blocked the activation of mTOR, and the accumulation of ROS by the ROS agonist blocked the activation of PI3K. In conclusion, these findings suggested that Se and Tau combination was better than either substance alone in protecting LPS-induced mammary inflammatory lesions by upregulating the PI3K/Akt/mTOR signaling pathway.

## 1. Introduction

Mastitis is an economically important pathology associated with reduced milk production, changes in milk composition, and quality, causing economic losses in the dairy industry all over the world [1–3]. The disease usually induces permanent and irreversible damage to milk-producing glandular tissues [4]. It is characterized by the inflammation of the mammary gland that occurs in response to various environmental and microbial predisposing factors [5]. Gram-negative bacteria are common pathogens that usually colonize dairy cows and represent a high-risk factor for mastitis. They are considered environmental mastitis pathogens, mainly including *Escherichia*, *Klebsiella*, *Enterobacter*, *Serratia*, *Pseudomonas*, and *Proteus*. Nowadays, antibiotic therapy is still the main method for the prevention and treatment of cow mastitis. However, the problem of antibiotic residue and antimicrobial resistance, in addition to the impact of antibiotic abuse on public health, leads to many restrictions on uncontrolled antibiotic therapy in the dairy sector worldwide [6, 7]. This scenario has made search for a novel and realistic prevention or treatment approach necessary. Recently, the combination or alone of natural molecules or derivatives with antibiotics to prevent or treat mastitis in dairy cows has attracted increasing attention [8–10].

Oxidative stress plays a pivotal role in several pathological events associated with animal production, including mastitis [11, 12]. Recent evidences have shown that dietary supplementation with antioxidant natural compounds, such as hydroxytyrosol [5], quercetin [13], moringa extract [14], and nanocurcumin [15], has shown protective action and improvement of dysfunctional inflammatory against in cows with mastitis. Selenium (Se) is an essential nutrient in animals and humans. Se supplementation, such as feeding Se yeast (SY), could reduce the incidence of breast infections in dairy cows [16, 17]. Selenomethionine (SeMet), the main form of organic Se in SY, possesses the anti-inflammatory and antioxidant effects [18–21]. Although the protective effects of SeMet against mastitis have been recognized [22, 23], they are not widely used in feed due to its high cost. Taurine (Tau), the major natural free amino acid in mammals, is widely available and less expensive. Tau has been reported to regulate the inflammatory response in mammary epithelial cells infected by *Streptococcus uberis* [24, 25]. However, it is not clear whether it can achieve the same effects as Se or combination with Se to produce a better effect in the prevention of mastitis or lower feed costs.

In this study, pregnant mice and bovine mammary epithelial cells (BMECs) were, respectively, chosen as an *in vivo* and *in vitro* model to explore the effects and the underlying mechanisms of SeMet and Tau alone or in a combination on LPS-induced mammary inflammatory lesions. Our findings will provide a new insight into using a nutritional approach for prevention of cow mastitis.

## 2. Materials and Methods

### 2.1. Animal Experiments

ICR female mice (7 days of gestation) were provided by the Center of Laboratory Animals, Yangzhou University (Yangzhou, China). The SeY (selenium content =1000 mg/kg) was purchased from Angel Yeast Co., Ltd. (Hubei, China), which was added to the diet in a premix form (completely replaced sodium selenite, selenium content = 0.3 mg/kg feed) by Xietong Medical Bioengineering Co., Ltd. (Jiangsu, China). The taurine (Tau) and lipopolysaccharide (LPS, from *Escherichia coli* 055: B5) were purchased from Sigma-Aldrich (St. Louis, MO, U.S.A.).

The experimental procedures were carried out under the approval of the ethical regulations of Nanjing Agricultural University (Permission number: NJAU.No20200303092). The mice were acclimatized for 1 week prior to experiments. On the 14^th^ day of the gestation, the mice were randomly divided into five groups (six mice each): a control group, a model group, a SeY group, a Tau group, and a SeY+Tau group. Over the entire experimental period, the mice of the SeY group and SeY+Tau group were fed a basic diet with SeY every day; the mice of the Tau group and SeY+Tau group were gavaged with Tau every day; other groups were replaced with equivalent amounts of PBS; the mice of control group and model group were fed a basic diet. On the 7^th^ day of the lactation, besides the control group, 50 *μ*L LPS (0.2 mg/mL) was injected into each nipple of mice in the remaining four groups. Serum and breast tissue were collected after 24 h.

### 2.2. Histopathological Examination

Mammary glands of mice were fixed in 4% formaldehyde solution, cut into paraffin sections, and stained according the protocol. Histological assessment was performed randomly using a scoring system according to the previous study [26]. Scores were given randomly: 0 for noninjury; 1 for mild injury; 2 for moderate injury; 3 for severe injury; and 4 for extreme severe injury.

### 2.3. Cell Culture

BMECs used in this experiment were purchased from Tongpai Biological Technology Co., Ltd. (Shanghai, China), and the cell line establishment methods were previously described by Zhao et al. [27]. Briefly, the 0.25% trypsin and 0.15% trypsin plus 0.02% EDTA were used to separate epithelial cells and fibroblasts of parenchymal tissues picked from a midlactation Holstein dairy cow mammary tissue according to their different sensitivity to trypsin. The epithelial origin and purity of the BMEC were assessed by immunofluorescence for cytokeratin 18, an epithelial-cell-specific marker (Figure S1). Cells were incubated in RPMI-1640 sterile media (Gibco, Paisley, Scotland, U.K.) containing 10% fetal bovine serum (FBS, Gibco) and 100 U·mL^−1^ each of penicillin and streptomycin (Gibco) at 37°C with 5% CO_2_. The Tau, LPS (from *Escherichia coli* 055: B5), selenomethionine (SeMet), and dimethyl sulfoxide (DMSO) were purchased from Sigma-Aldrich (St. Louis, MO, U.S.A.).

### 2.4. Cell Viability Assay

The cytotoxicity of SeMet and Tau alone or in combination to BMECs was detected by MTT and lactate dehydrogenase (LDH) release assays. Briefly, cells in 96-well plates (5 × 10^3^ cells/well) were incubated with SeMet (0, 1, 2, 4, 8, 16, and 32 *μ*M) and Tau (0, 5, 10, 20, 40, 60, and 80 mM) individually or in combination for 48 h. Then, the cell culture medium was determined by LDH kit (Jiancheng, China). And the cells were treated with 15 *μ*L/well of MTT (Sigma-Aldrich) for 3 h and dissolved with 100 *μ*L/well of DMSO. The optical density (OD) values were determined using a microplate reader (Bio-Rad, Hercules, CA, USA), respectively. There are five repetitions per sample for each assay.

### 2.5. Quantitative Real-Time PCR (qRT-PCR)

Cells in 12-well plates (8 × 10^4^ cells/well) were pretreated with SeMet and/or Tau for 36 h and then were stimulated with LPS (10 *μ*g·mL^−1^) for 12 h [28, 29]. Every treatment batch consisted of 3 parallels. The total RNA was extracted following the manufacturer's instructions using the RNAiso Plus kit (TaKaRa). Then, the RNA was reverse transcription to cDNA using High Capacity cDNA Reverse Transcription Kit (TaKaRa). Gene expression was performed using SYBR Premix Ex TaqII (TaKaRa) and the ABI Prism Step One Plus detection system (Applied Biosystems, Foster City, CA, USA). The relative mRNA levels were calculated using the *Δ*cycle threshold (*Δ*Ct) method, with *β*-actin as the housekeeping gene. All primers were synthesized by Invitrogen and are listed in Table 1.

### 2.6. Oxidative Stress Detection

The nitric oxide (NO) production, the activity of superoxide dismutase (SOD), the malondialdehyde (MDA) levels, and the total antioxidant capacity (T-AOC) were detected by commercially available kits (Jiancheng) according to the manufacturer's instructions. For the detection of the intracellular reactive oxygen species (ROS), cells were firstly seeded on the surface of 20 mm diameter round coverslips (WHB, Nanjing, Jiangsu, China) in 12-well plates. After being treated accordingly, the cell culture medium was discarded. Then, cells were stained at 37°C with 2′,7′-dichlorofluorescein diacetate (DCFH-DA; Sigma-Aldrich) for 20 min. Zeiss LSM 710 META confocal system of laser scanning confocal microscopy (Zeiss, Oberkochen, German) was used to scan cells. In the microscope, intracellular ROS were labeled with green fluorescence.

### 2.7. Apoptosis Assay

The apoptosis rate was determined using Annexin V-FITC/PI staining by flow cytometry. After treatments, cells were digested and collected into 1.5 mL tubes and then stained using Annexin V-FITC/PI apoptosis detection kit (Vazyme Biotech, Nanjing, Jiangsu, China). After washing three times, 10,000 cells per tube were resuspended and collected by flow cytometry (FACS Calibur, BD Biosciences, San Jose, CA, USA). The ratio of Bcl-2 and Bax was determined based on the detected mRNA expressions.

### 2.8. Western Blotting

The 12% sodium dodecyl sulfate-polyacrylamide gel electrophoresis (SDS-PAGE, Solarbio, Beijing, China) was used to separate an equal amount of proteins (30 *μ*g). Extracted total proteins were transferred onto polyvinylidene difluoride (PVDF) membranes and incubated with 5% Albumin Bovine (BSA, Solarbio) for 2 h. Then, the membranes were placed overnight with the primary antibodies of *β*-actin (Cell Signaling Technology 4970S, 1 : 2000), PI3K (Cell Signaling Technology 4257S, 1 : 1000), total (t)-AKT (Cell Signaling Technology 4691S, 1 : 1000), phosphorylated (p)-AKT (Cell Signaling Technology 4060S, 1 : 1000), t-mTOR (Abcam ab2732, 1 : 1000), or p-mTOR (Abcam ab109268, 1 : 1000) at 4°C. After washing with 1 × TBST three times, the membranes were incubated with the second antibody of horseradish peroxidase- (HRP-) conjugated goat anti-rabbit IgG (Cell Signaling Technology) for 1 h. The expected protein bands were visualized by ECL Plus Detection Reagent Kit (Biosharp) and detected using Image Quant LAS 4000 (GE Healthcare Life Sciences, USA).

### 2.9. Statistical Analysis

The experiments were performed in three replicates independently for each group, and the values are presented as the mean ± standard error (SE). Data analyses were performed statistically with SPSS 18.0 (SPSS Inc., Chicago, IL, USA) and GraphPad Prism (version 5.0, Graph Pad Software Inc., San Diego, CA) by Student's *t*-test and one-way analysis of variance (ANOVA). *P* < 0.05 was significant difference, and *P* < 0.01 was extremely significant difference.

## 3. Results

### 3.1. Supplementation of SeY and Tau Alone or in Combination in Feed Alleviated the LPS-Induced Mammary Inflammatory Damage in Mice

**Figure 1:**
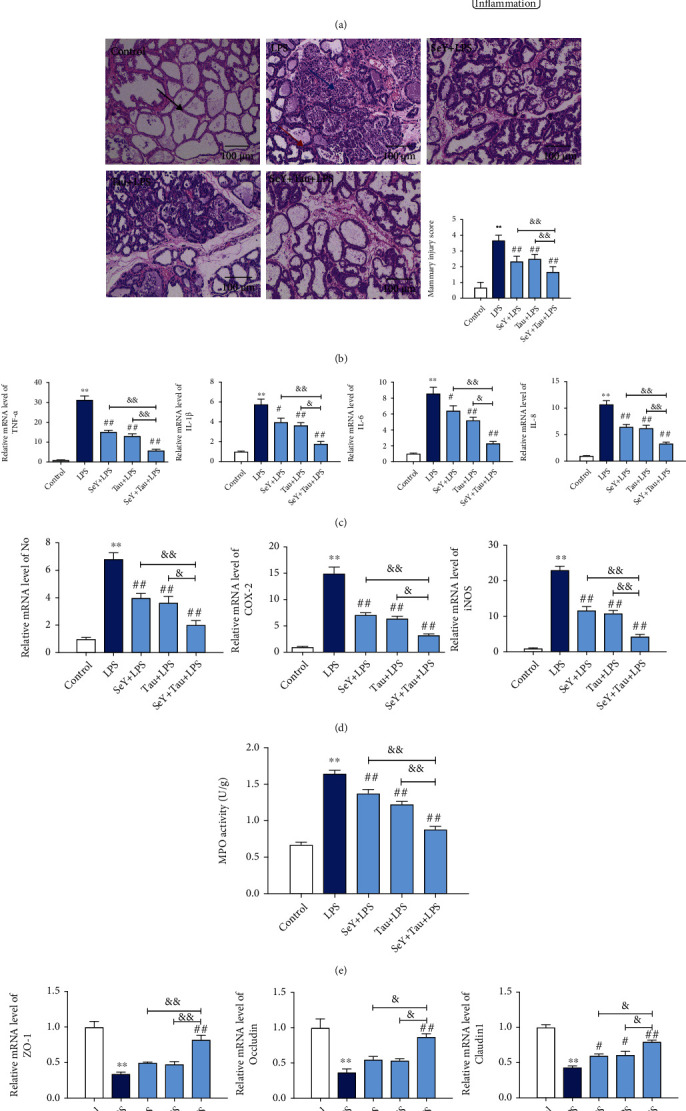
Effects of SeY and Tau alone or in combination in feed on the LPS-induced inflammatory damage of the mammary tissues in mice. (a) The design of animal experiments. (b) Histopathological changes of the mammary tissues (100x). The black, blue, and red arrows were the normal tissues, the infiltration of inflammatory cells, and the hyperplastic of the alveolar wall, respectively. (c) The levels of proinflammatory cytokines TNF-*α*, IL-1*β*, IL-6, and IL-8. (d) The levels of inflammatory mediators NO, COX-2, and iNOS. (e) The MPO activity. (f) The levels of tight junction proteins ZO-1, Occludin, and Claudin1. All results are presented as the means ± SEM of three independent experiments (*n* = 6). Significance compared with control, ^∗^*P* < 0.05 and^∗∗^*P* < 0.01. Within the LPS-treated groups, significance compared with the LPS group, ^#^*P* < 0.05, ^##^*P* < 0.01, ^&^*P* < 0.05, and ^&&^*P* < 0.01.

To determine whether the combined use of Se and Tau has a protective effect on animal mastitis, pregnant mice and LPS were used to establish an *in vivo* mastitis model. ICR gestation mice were fed with SeY and/or Tau from the 14^th^ day of gestation, and LPS was injected into the mammary glands to cause mastitis on the 7^th^ day of lactation (Figure 1(a)). Compared with the control group, the histopathological results showed that the structure of mammary acini in the LPS group was significantly damaged, and the acinar wall was significantly thickened, associated with a large number of inflammatory cellular infiltration (Figure 1(b)). The addition of SeY and Tau alone could alleviate, to some extent, the inflammatory damage caused by LPS, while the combination of SeY and Tau could significantly improve the inflammatory damage so that the structure of mouse mammary acinus was more complete and the inflammatory infiltrating cells were significantly reduced (Figure 1(b)). In addition, LPS-induced increases in the proinflammatory cytokines (TNF-*α*, IL-1*β*, IL-6, and IL-8), the inflammatory mediators (NO, iNOS, and COX-2), and the MPO activity were significantly inhibited by SeY and Tau alone or in combination, but the combination of SeY and Tau provided a better result (Figures 1(c)–1(e)). Then, the effects of SeY and Tau alone or in combination on blood-milk barrier function were investigated. The results showed that the levels of tight junction protein (ZO-1, Occludin, and Claudin1) were significantly decreased by LPS, but significantly restored by the addition of Se and Tau alone or in combination, and the effect of the combination of SeY and Tau was more obvious (Figure 1(f)).

### 3.2. Supplementation of SeY and Tau Alone or in Combination in Feed Activated the PI3K/Akt/mTOR Signaling Pathway of the Mammary Tissue in Mice

**Figure 2:**
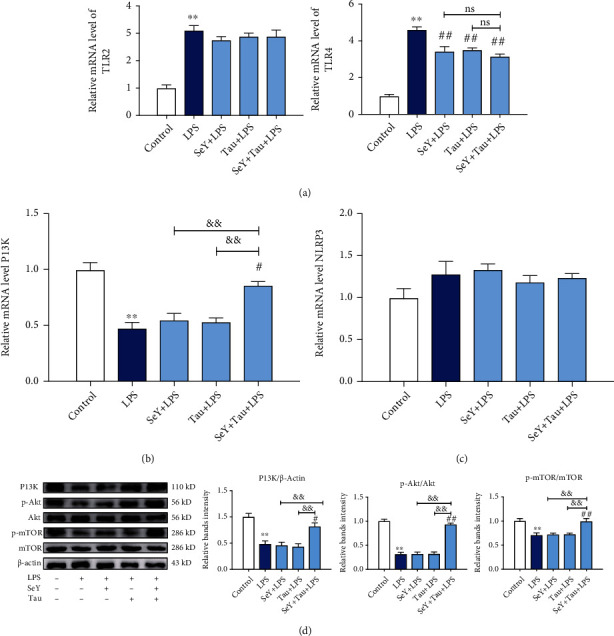
Effects of SeY and Tau alone or in combination in feed on the inflammation related signaling pathways of the mammary tissues in mice. (a–c) The mRNA levels of TLR2, TLR4, NLRP3, and PI3K. (d) The protein levels of PI3K pAKT and pmTOR. All results are presented as the means ± SEM of three independent experiments (*n* = 6). Significance compared with control, ^∗^*P* < 0.05 and ^∗∗^*P* < 0.01. Within the LPS-treated groups, significance compared with the LPS group, ^#^*P* < 0.05, ^##^*P* < 0.01, ^&^*P* < 0.05, and ^&&^*P* < 0.01.

TLRs, NLRP3, and PI3K signaling pathways are involved in the inflammatory response and cell survival of many cells; therefore, we examined the mRNA levels of TLR2, TLR4, NLRP3, and PI3K to determine the underlying mechanism of SY and Tau alone or in combination implicated in reducing mammary inflammatory damage. The results showed that SY and Tau alone or in combination significantly inhibited LPS-activated TLR4, but there was no significant difference between SY/Tau alone and in combination groups (Figure 2(a)). Compared with SY or Tau group, the combination group significantly activated LPS-suppressed PI3K (Figure 2(b)). TLR2 and NLRP3 were not involved in LPS-induced mastitis (Figures 2(a) and 2(c)). The western blotting results further showed that the abundance of PI3K and its downstream Akt/mTOR pathway proteins in mice fed with SeY and Tau in combination were significantly increased compared with the SeY+LPS group and Tau+LPS group (Figure 2(d)). All *in vivo* results concluded that the combination of SeY and Tau could significantly alleviate LPS-stimulated mammary inflammatory damage in mice, and this effect might be related to the activation of PI3K/Akt/mTOR signaling pathway.

### 3.3. Effects of SeMet and Tau Alone or in Combination on Cell Activity of BMCs

**Figure 3:**
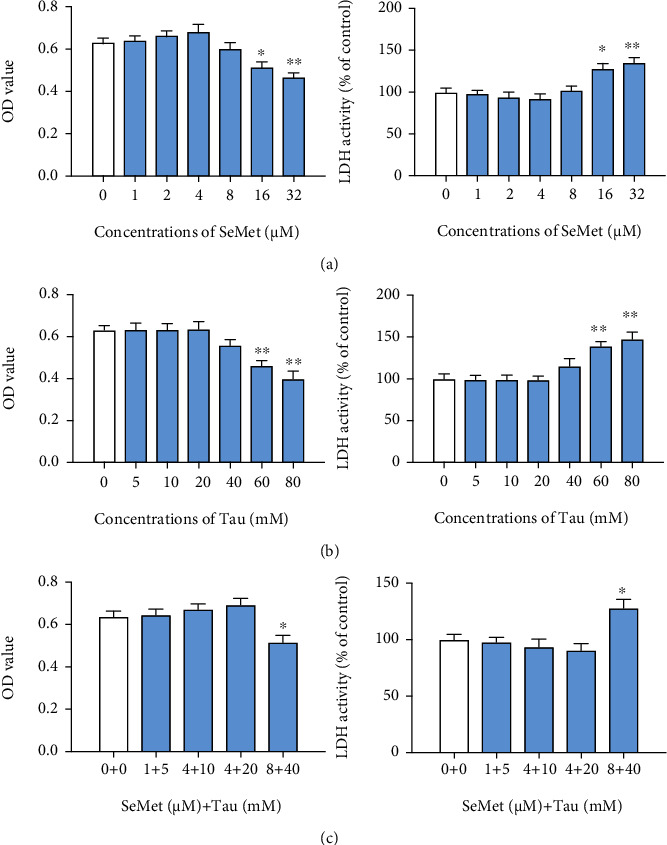
Effects of SeMet and Tau alone or in combination on the cell viability of BMECs. The cell viabilities of BMECs were detected by MTT and LDH assays processed according to the procedures described in Materials and Methods. (a) The MTT and LDH results of SeMet. (b) The MTT and LDH results of Tau. (c) The MTT and LDH results of SeMet and Tau in combination. All results are presented as the means ± SEM (*n* = 3). Statistical significance compared with control, ^∗^*P* < 0.05 and ^∗∗^*P* < 0.01.

To detect cytotoxicity of -SY and Tau alone or in combination to BMECs, cells were cultured in the presence of SeMet (0, 1, 2, 4, 8, 16, or 32 *μ*M; the main form of organic selenium in SY), Tau (0, 5, 10, 20, 40, 60, or 80 mM), or SeMet+Tau (0 + 0, 1 *μ*M + 5 mM, 2 *μ*M + 10 mM, 4 *μ*M + 20 mM, and 8 *μ*M + 40 mM) for 48 h, and the cell viability was measured by MTT and LDH assays. As shown in Figure 3, there was a significant decrease in cell viability of BMECs which treated with 16-32 *μ*M SeMet (Figure 3(a)), 60-80 mM Tau (Figure 3(b)), and 8 + 40 group (Figure 3(c)). So, SeMet at 0, 1, 2, 4, and 8 *μ*M, Tau at 0, 5, 10, 20, and 40 mM and the combination of 4 *μ*M SeMet + 20 mM Tau were used in subsequent experiments.

### 3.4. SeMet and Tau Alone or in Combination Ameliorated the Inflammation and Blood-Milk Barrier Dysfunction of BMECs Induced by LPS

**Figure 4:**
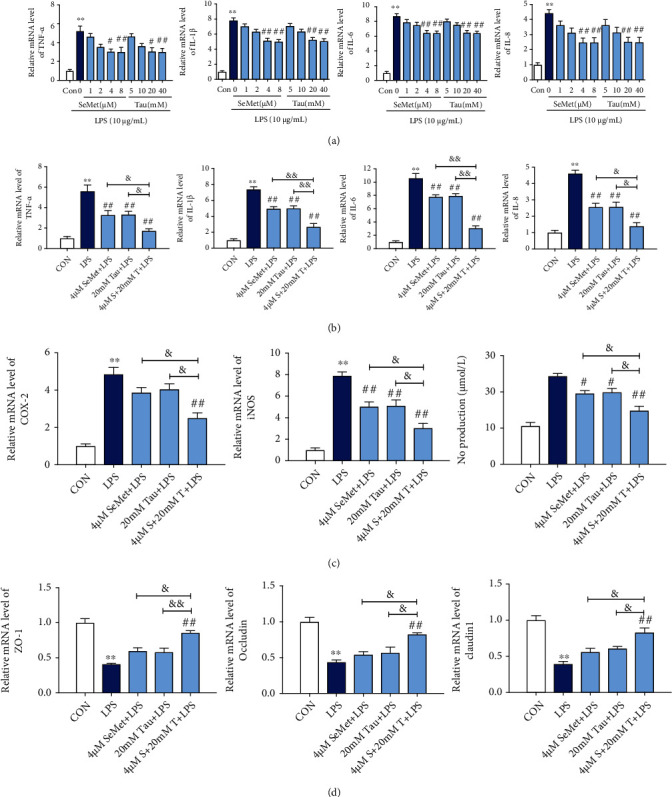
Effects of SeMet and Tau alone or in combination on the inflammation and blood-milk barrier in the presence of LPS in BMECs. (a, b) The mRNA levels of pro-inflammatory cytokines TNF-*α*, IL-1*β*, IL-6, and IL-8. (c) The levels of inflammatory mediators NO, iNOS, and COX-2. (d) The mRNA levels of tight junction proteins ZO-1, Occludin, and Claudin1. All results are presented as the means ± SEM (*n* = 3). Significance compared with control, ^∗^*P* < 0.05 and ^∗∗^*P* < 0.01. Within the LPS-treated groups, significance compared with LPS group, ^#^*P* < 0.05, ^##^*P* < 0.01, ^&^*P* < 0.05, and ^&&^*P* < 0.01.

To detect the effects of SeMet and Tau alone or in combination on the LPS-induced inflammation in BMECs, mRNA levels of proinflammatory cytokines and inflammatory mediators were detected by qRT-PCR. In the presence of LPS, the mRNA levels of proinflammatory cytokines (TNF-α, IL-1*β*, IL-6, and IL-8), were significantly increased in BMECs (*P* < 0.01), but these increases were markedly reduced by SeMet and Tau alone or in combination, and the effect of the combination group was better than that observed in the other groups (Figures 4(a) and 4(b)). The changes of inflammatory mediators (NO, iNOS, and COX-2) were consistent with those of proinflammatory cytokines (Figure 4(c), *P* < 0.05). For the blood-milk barrier, the mRNA levels of tight junction protein (ZO-1, Occludin, and Claudin1) were determined. As shown in Figure 4(d), the decreased mRNA levels of ZO-1, Occludin, and Claudin1 by LPS were remarkably increased by the combined treatments of SeMet and Tau. These results indicated that the effects of the combination of SeMet and Tau on the LPS-induced inflammation and blood-milk barrier dysfunction in BMECs were better than that of alone.

### 3.5. SeMet and Tau Alone or in Combination Ameliorated the Death of BMECs Induced by LPS

**Figure 5:**
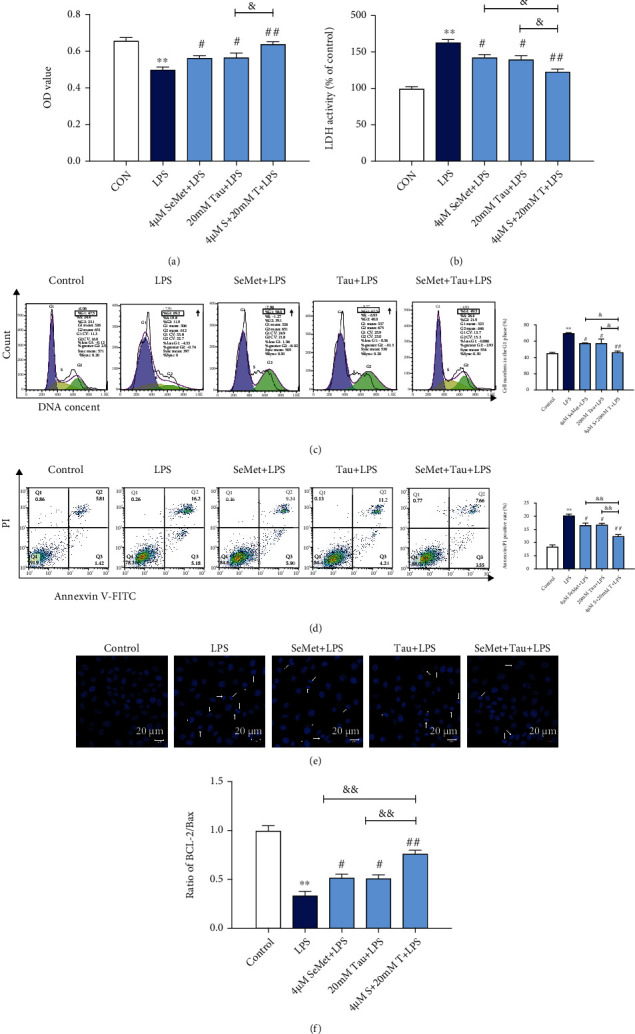
Effects of SeMet and Tau alone or in combination on the cell proliferation and apoptosis of BMECs. The cell viability (a), the membrane permeability (b), the cell cycle (c), the formation of apoptotic bodies (d), the apoptosis rate (e), and the ratio of Bcl-2/Bax (f) of BMECs were detected. All results are presented as the means ± SEM (*n* = 3). Significance compared with control, ^∗∗^*P* < 0.01. Within the LPS-treated groups, significance compared with the LPS group, ^#^*P* < 0.05, ^##^*P* < 0.01, ^&^*P* < 0.05, and ^&&^*P* < 0.01.

To detect the effects of SeMet and Tau alone or in combination on the death of BMECs induced by LPS, cell proliferation, and cell membrane permeability were measured by MTT, cell cycle, and LDH assays. Stimulation of BMECs with LPS significantly inhibited the proliferation (Figure 5(a)), increased the cell membrane permeability (Figure 5(b)), and delayed the transition from G1 to S phase (Figure 5(c)). As expected, these changes were recovered more significantly in the combined group than in the SeMet -or Tau alone groups (Figures 5(a)–5(c)). The effects of SeMet and Tau alone or in combination on LPS-induced apoptosis in BMECs were then assessed using Hoechst33258 staining, Annexin V/PI, and qRT-PCR. Compared to the control group, LPS at 10 *μ*g·mL^−1^induced cells concentrated and fractured (Figure 5(d)), increased apoptosis rate (Figure 5(e), *P* < 0.01), and decreased the ratio of Bcl-2 and Bax (Figure 5(f), *P* < 0.01). Treatment with 4 *μ*M SeMet and 20 mM Tau alone or in combination decreased the apoptotic cell numbers (Figure 5(d)), the apoptosis rate (Figure 5(e), *P* < 0.01) and increased the ratio of Bcl-2 and Bax (Figure 5(f), *P* < 0.05), and the combination group resulted in a better effect. These results indicated that SeMet and Tau in combination afforded more significant protection against LPS-induced cell death than SeMet or Tau alone did in BMECs.

### 3.6. SeMet and Tau in Combination Restored the PI3K/Akt/mTOR Signaling Pathway Inhibited by LPS in BMECs

**Figure 6:**
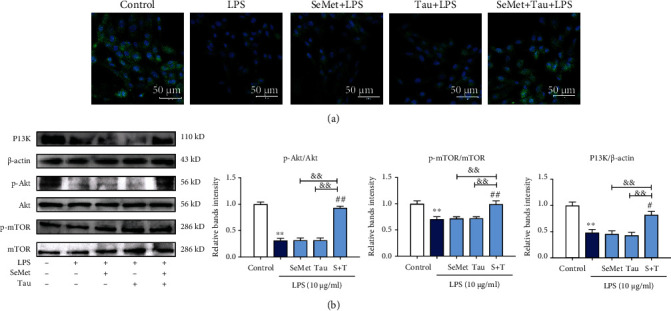
Effects of SeMet and Tau alone or in combination on the expressions of PI3K, p-Akt, and p-mTOR in LPS-stimulated BMECs. (a) The expression of PI3K was examined by immunofluorescence. (b) The protein expressions of PI3K, p-Akt/Akt, and p-mTOR/mTOR were examined by western blotting. All results are presented as the means ± SEM (*n* = 3). Significance compared with control, ^∗^*P* < 0.05 and^∗∗^*P* < 0.01. Within the LPS-treated groups, significance compared with LPS group, ^#^*P* < 0.05, ^##^*P* < 0.01, ^&^*P* < 0.05, and ^&&^*P* < 0.01.

To determine the underlying mechanism of SeMet and Tau in combination reduced mammary inflammatory lesions, the protein abundance of PI3K in LPS-treated BMECs was detected. The indirect immunofluorescence (IFA) results showed that the combination of SeMet and Tau increased the protein expression of PI3K (Figure 6(a)). The western blotting results were consistent with the IFA (Figure 6(b), *P* < 0.05). In addition, the phosphorylations of Akt and mTOR, the protein abundance of PI3K downstream pathway proteins, were significantly increased by the combination of SeMet and Tau compared with the LPS group (Figure 6(b), *P* < 0.05). Both SeMet or Tau alone failed to increase these proteins. These results reported that the combination of SeMet and Tau could restore the PI3K/Akt/mTOR signaling pathway inhibited by LPS in BMECs.

### 3.7. The Activation of PI3K/Akt/mTOR Was Responsible for the Combined Protection of SeMet and Tau against LPS-Induced Inflammatory Damage in BMECs

**Figure 7:**
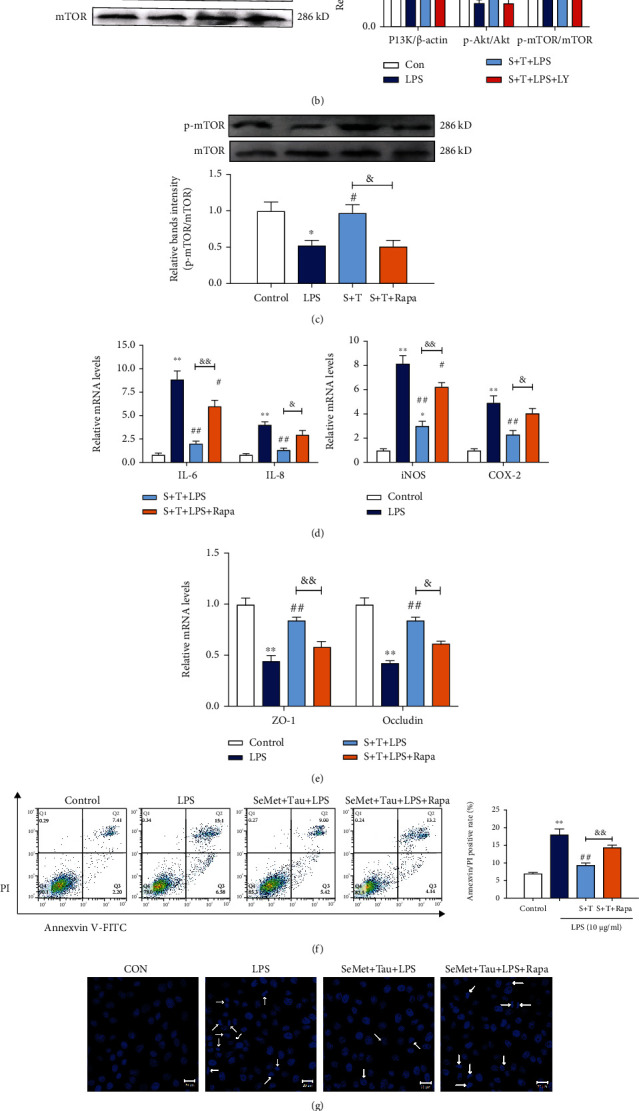
Effects of LY294002 and Rapa on the activation of p-Akt/p-mTOR in LPS-stimulated BMECs. (a) The expression of PI3K in the presence of LY294002 was examined by immunofluorescence. (b) The protein expressions of PI3K, p-Akt/Akt, and p-mTOR/mTOR in the presence of LY294002 were examined by western blotting. (c) The protein levels of p-mTOR/mTOR in the presence of Rapa were examined by western blotting. (d) The mRNA levels of IL-6, IL-8, iNOS, and COX-2. (e) The mRNA levels of ZO-1 and Occludin. (f) The apoptosis rate. (g) The formation of apoptotic bodies. (h) The ratio of Bcl-2/Bax. All results are presented as the means ± SEM (*n* = 3). Significance compared with control, ^∗^*P* < 0.05 and^∗∗^*P* < 0.01. Within the LPS-treated groups, significance compared with LPS group, ^#^*P* < 0.05, ^##^*P* < 0.01, ^&^*P* < 0.05, and ^&&^*P* <0.01.

An inhibitor of PI3K, LY294002 [30], was first used to verify whether mTOR is mediated by PI3K. As shown in Figures 7(a) and 7(b), PI3K was inhibited by LY294002, while the phosphorylation of Akt and mTOR was also inhibited (*P* < 0.05), indicating that mTOR was the downstream of the PI3K/Akt pathway. Rapamycin (Rapa, an inhibitor of mTOR) [31] was used to further determine whether the PI3K/Akt/mTOR pathway was involved in the combined protective effects of SeMet and Tau against LPS-induced inflammatory injury in BMECs. As shown in Figure 7(c), Rapa downregulated p-mTOR activated by SeMet and Tau in combination (*P* < 0.05). At the same time, Rapa counteracted the protection of SeMet and Tau in combination against LPS-induced inflammation, blood-milk barrier dysfunction, and apoptosis in BMECs to some extent as demonstrated by significant increases in the mRNA levels of IL-6/IL-8/iNOS/COX-2 (Figure 7(d)) and ZO-1/Occludin (Figure 7(e)), the apoptotic rate (Figure 7(f)), the number of apoptotic bodies (Figure 7(g)), and the ratio of Bcl-2 and Bax (Figure 7(h)). These data suggested that the combination of SeMet and Tau protected BMECs against LPS-induced inflammatory damage by activating PI3K/Akt/mTOR signaling pathway.

### 3.8. SeMet and Tau in Combination Blocked the Inhibitory Effect of LPS-Produced ROS on PI3K in BMECs

**Figure 8:**
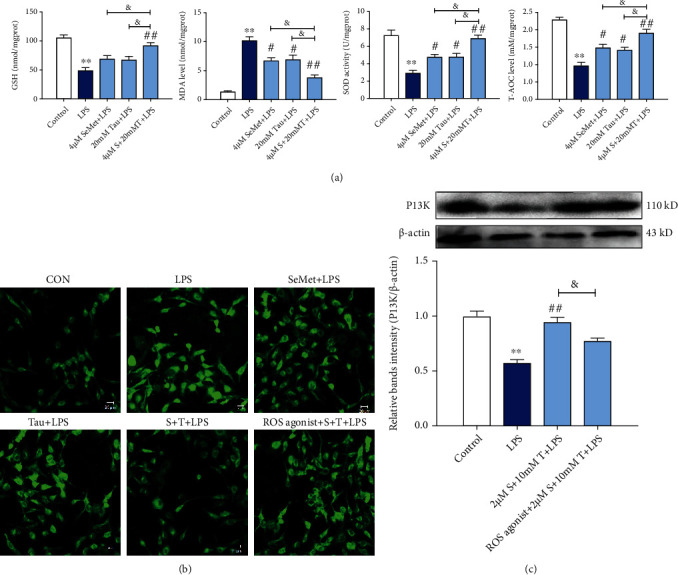
Effects of SeMet and Tau alone or in combination on oxidative stress and ROS agonist on the activation of PI3K in LPS-stimulated BMECs. (a) The determination of GSH contents, SOD activity, MDA level, and T-AOC level. (b, c) The determination of intracellular ROS and PI3K expression in the presence of ROS agonist. All results are presented as the means ± SEM (*n* = 3). Significance compared with control, ^∗^*P* < 0.05 and^∗∗^*P* < 0.01. Within the LPS-treated groups, significance compared with the LPS group, ^#^*P* < 0.05, ^##^*P* < 0.01, and ^&^*P* < 0.05.

To elucidate how SeMet and Tau in combination activated the LPS-suppressed PI3/Akt/mTOR signaling pathway, the levels of oxidative stress in BMECs were measured. As indicated in Figures 8(a) and 8(b), the production of GSH, the activity of SOD, and the level of T-AOC were significantly decreased, and the level of MDA and intracellular ROS were considerably increased in the LPS group (*P* < 0.01) compared with the control group. But the aforementioned changes induced by LPS were significantly reversed by the SeMet and Tau alone and in combination, and the combination had a more significant effect on the changes than alone (Figures 8(a) and 8(b)). Moreover, when the inhibition of ROS by the combination of SeMet and Tau was reincreased by the ROS agonist (Figure 8(b)), the activation of PI3K in BMECs was significantly counteracted (Figure 8(c), *P* < 0.05). The results showed that the combination of SeMet and Tau was more effective in enhancing the cellular antioxidant capacity in BMECs than a single supplementation of SeMet or Tau and blocked the inhibitory effect of LPS on PI3K by scavenging ROS in BMECs.

## 4. Discussion

Preventing and treating dairy cow mastitis are complex and costly [32, 33]. Several studies have been conducted to find new alternatives to replace or assist antibiotics or vaccine control. In this study, we used LPS-stimulated BMECs and pregnant mice to establish the inflammatory model of bovine mastitis *in vitro* [34, 35] and *in vivo* [36], and found that the combined supplementation of Se and Tau was more effective than Se or Tau alone in alleviating mammary inflammatory lesions induced by LPS via upregulation of the PI3K/Akt/mTOR signaling pathway.

The inflammatory response is a process to activate the immune system caused by stimuli, including infection, which is mediated by multiple signal molecules produced by types of cells [37, 38]. Stimulated by LPS, the surface secretions of *E. coli*, BMECs reacted to inflammatory reaction and produced proinflammatory cytokines such as IL-1*β*, IL-6, and IL-8 [34, 39]. Previous literature has shown that these cytokines could interact with each other in cells, which results in tissue damage in mammary gland [40, 41]. It has been reported that the proinflammatory enzymes iNOS and COX-2, as well as the main activation product of the iNOS, play critical roles in mediating inflammation [42]. Abnormal production of them can lead to cell damage even tissue injury. In this study, our results indicated that LPS could lead to drastic increases of proinflammatory cytokines (TNF-*α*, IL-1*β*, IL-6, and IL-8) and inflammatory mediators (iNOS, COX-2, and NO) in the mammary tissue of mice and BMECs, which were consistent with previous reports. Both SeMet and Tau alone and in combination could significantly decrease these cytokines and mediators. Moreover, a much stronger decrease was observed after being treated by the combination of SeMet and Tau. The mammary epithelium plays a vital role in forming a physical and interactive barrier between the blood and milk [43]. Destruction of the blood–milk barrier aggravates bacterial infection and promotes the development of inflammation [44]. Xu et al. demonstrated that IL-1*β* induced blood-milk barrier damage in BMECs [45]. In this study, SeMet and Tau combination significantly LPS-induced apoptosis and cell cycle, which is consistent with the protective effects of sodium propionate on the blood-milk barrier integrity reported by Ali et al. [46].

Previous studies showed that mTOR usually play an important role in inflammatory response including negatively regulating LPS-induced inflammation *in vitro* as the downstream of the PI3K/Akt signaling pathway [47–49]. The PI3K/Akt/mTOR signaling pathway is considered to be critical to cell growth, proliferation, and survival in mammalian cells, including mammary epithelial cells, and recently has also emerged to be involved in the regulation of inflammatory response [34, 50, 51]. Consistent with a previous report, in this study, we found that LPS markedly restrained the protein abundanceof PI3K, p-Akt, and p-mTOR *in vitro* and *vivo*, and then the cotreatment with Se and Tau reversed that. When BMECs were pretreated with the mTOR inhibitor Rapa, the combined protection of SeMet and Tau against LPS-stimulated inflammatory injury was abolished. PI3K, which is a key player in various cellular responses, triggers Akt, an intracellular serine/threonine kinase, then consequently activates mTOR [52]. When BMECs were pretreated with the PI3K inhibitor LY294002, PI3K protein abundance, p-Akt, and p-mTOR were all depressed, suggesting that mTOR was also downstream of the PI3K/Akt pathway in our study.

Relationships between oxidative stress, apoptosis, and inflammation have been documented by many authors. Current studies have proved the importance of excessive ROS in inducing various cell apoptosis by the damage of lipids, proteins, and DNA in cells [53, 54]. Our study demonstrated that the increase of ROS participated in LPS-induced apoptosis of BMECs, which was consistent with a previous study [46, 55]. Moreover, excessive generation of ROS and the deficiency of antioxidants can result in the occurrence of oxidative stress which contributes to cell inflammation by inducing several signal molecules [38]. The accumulation of ROS could inhibit expression of PI3K [56]. We found the combination of SeMet and Tau significantly eliminated the ROS and inhibited the LPS-induced mammary inflammatory injury but was abolished by ROS agonist, suggesting that elimination of ROS by SeMet and Tau in combination and Tau was responsible for the inhibition of PI3K.

All the experiments indicated that treatment of Se or Tau alone could attenuate LPS-stimulated mammary inflammatory damage *in vitro* and *vivo*, and the effects of combined treatment of Se and Tau were better. In addition, Se and Tau in combination played a beneficial role via activating PI3K/Akt/mTOR signaling pathway, and the activation of the pathway was mainly attributed to the elimination of ROS by Se and Tau in combination. Our works proved that it was feasible to prevent dairy cow mastitis by the combined application of Se and Tau in theory.

## Figures and Tables

**Table 1:** Primer sequences.

Genes	Forward primer 5′-3′	Reverse primer 5′-3′
*In vitro*		
IL-1*β*	GGCAACCGTACCTGAACCCA	CCACGATGACCGACACCACC
IL-6	CCTTCACTCCATTCGCTGTCT	TCCTGATTTCCCTCATACTCG
IL-8	ATGACTTCCAAGCTGGCTGTT	GGTTTAGGCAGACCTCGTTTC
iNOS	GGACTTGGCTACGGAACTGG	GGTGAAGCGTGTCTTGGAAA
COX-2	GGCGATGAGCAGTTGTTCCA	TGCTGTACGTAGTCTTCAATCACAAT
Bax	GTGCCCGAGTTGATCAGGAC	CCATGTGGGTGTCCCAAAGT
Bcl-2	GAGTTCGGAGGGGTCATGTG	GCCTTCAGAGACAGCCAGGA
ZO-1	GACTTGTCAGCTCAGCCAGT	GGCTCCTCTCTTGCCAACTT
Occludin	TTCTTCAGGCGGAGACGGC	GATGACATGGCTGGTGTCAGTG
Claudin1	CCCGTGCCTTGATGGTGATT	AAGACAGCCATCCGCATCTT
*In vivo*		
TNF-*α*	ATGTCTCAGCCTCTTCTCATTC	GCTTGTCACTCGAATTTTGAGA
IL-1*β*	GCAACTGTTCCTGAACTCAACT	ATCTTTTGGGGTCCGTCAACT
IL-6	TTCTTGGGACTGATGCTGGT	AGACAGGTCTGTTGGGAGTG
iNOS	TGCCACGGACGAGACGGATAG	CTCTTCAAGCACCTCCAGGAACG
COX-2	GGTGCCTGGTCTGATGATGTATGC	GGATGCTCCTGCTTGAGTATGTCG
ZO-1	ACTCCCACTTCCCCAAAAAC	CCACAGCTGAAGGACTCACA
Occludin	ATTCCATCAGTTTCCTATCT	ACCAGGACCTTTCTTGAC
Claudin1	AGACCTGGATTTGCATCTTGGTG	TGCAACATAGGCAGGACAAGAGTTA

## Data Availability

The data used to support the findings of this study are included within the article.

## Abbreviations

BMECs: Bovine mammary epithelial cells
COX-2: Cyclooxygenase-2
LPS: Lipopolysaccharide
MDA: Malondialdehyde
NO: Nitric oxide
PVDF: Polyvinylidene difluoride
iNOS: Inducible nitric oxide synthase
SeMet: Selenomethionine
SDS-PAGE: Sodium dodecyl sulfate-polyacrylamide gel electrophoresis
SOD: Superoxide dismutase
T-AOC: Total antioxidant capacity
Tau: Taurine
ROS: Reactive oxygen species.

## Supplementary Materials

Supplementary Materials Figure S1: detection of the epithelial origin and purity of the BMECs by immunofluorescence for cytokeratin 18. All results are presented as the means ± SEM (*n* = 3).
