# Supplementary figures and images for: Selenium and Taurine Combination Is Better Than Alone in Protecting Lipopolysaccharide-Induced Mammary Inflammatory Lesions via Activating PI3K/Akt/mTOR Signaling Pathway by Scavenging Intracellular ROS
Source: Oxid Med Cell Longev. 2021 Dec 13;2021:5048375. doi: 10.1155/2021/5048375 (PMC8687852; doi:10.1155/2021/5048375)

DAPI

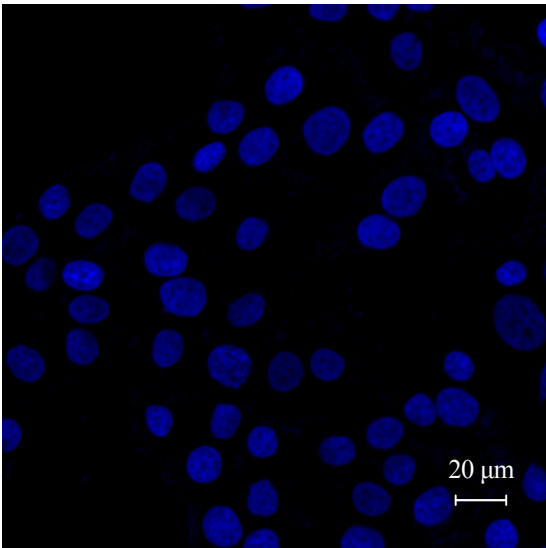

CK-18

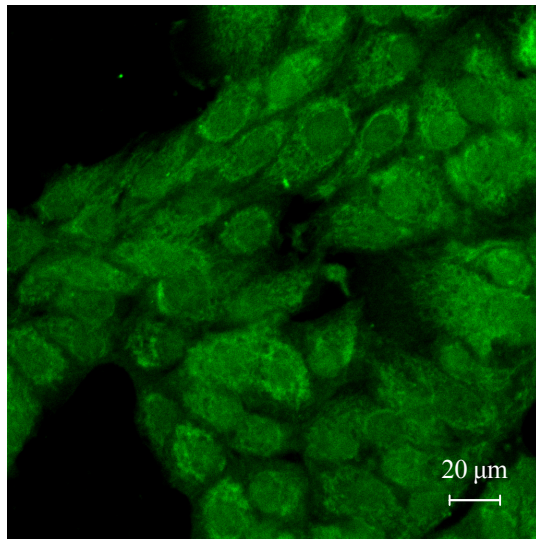

Merged

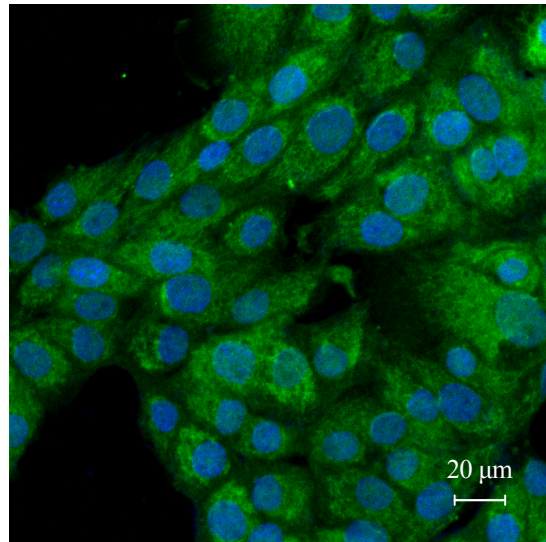

Supplement: Supplementary Materials — Figure S1: detection of the epithelial origin and purity of the BMECs by immunofluorescence for cytokeratin 18. All results are presented as the means ± SEM (n = 3). [file 5048375.f1.pdf]
